# Identification of Neuropeptides Using Long-Read RNA-Seq in the Swimming Crab *Portunus trituberculatus*, and Their Expression Profile Under Acute Ammonia Stress

**DOI:** 10.3389/fphys.2022.910585

**Published:** 2022-05-16

**Authors:** Daixia Wang, Xiaochen Liu, Jingyan Zhang, Baoquan Gao, Ping Liu, Jian Li, Xianliang Meng

**Affiliations:** ^1^ Key Laboratory of Aquatic Genomics, Ministry of Agriculture and Rural Affairs, Yellow Sea Fisheries Research Institute, Chinese Academy of Fishery Sciences, Qingdao, China; ^2^ Laboratory for Marine Fisheries Science and Food Production Processes, Qingdao National Laboratory for Marine Science and Technology, Qingdao, China; ^3^ Key Laboratory of Sustainable Development of Marine Fisheries, Ministry of Agriculture and Rural Affairs, Yellow Sea Fisheries Research Institute, Chinese Academy of Fishery Sciences, Qingdao, China

**Keywords:** long-read transcriptome, *Portunus trituberculatus*, neuropeptide, ammonia, crab

## Introduction

The swimming crab *Portunus trituberculatus* (*P.trituberculatus*) is widely distributed in estuary and coastal areas of temperate western Pacific Ocean (Dai et al., 1986), and comprises a large aquaculture industry in China with a production of 100,895 tons in 2020 (China Fishery Statistical Yearbook 2021). To satisfy the growing market demand, the crab aquaculture has been moving toward more intensive systems with higher feed inputs and stocking density. In intensive culture systems, ammonia (throughout this paper, the term “ammonia” refers to the sum of NH_3_ and NH_4_^+^), mainly derives from the decomposition of leftover feeds and the excretion of cultured crabs, has been recognized the major limiting factor (Pan et al., 2018; Si et al., 2019; Zhang et al., 2021). Ammonia is a toxic molecule to aquatic animals, including the swimming crab (Liu et al., 2015; Pan et al., 2018). Recent studies have showed that ammonia exposure can disturb immune response (Romano and Zeng, 2013), which causes tissue injury (Si et al., 2019; Lu et al., 2022) and even death of *P. trituberculatus* (Liu et al., 2015; Zhao et al., 2020). Due to the increasing concerns on ammonia toxicity, several studies have been conducted on ammonia detoxification strategies in crustaceans. These studies found that the swimming crab can defend against environmental ammonia through several compensatory mechanisms, for example, ammonia excretion *via* its transporters in branchial epithelium, ammonia conversion into non-toxic or less toxic substances, and decreasing the rate of metabolic generated ammonia (Mykles et al., 2010; Henry et al., 2012; Liu et al., 2015; Pan et al., 2018; Zhang et al., 2021). In fact, strategies of ammonia detoxification have been identified, but the regulation mechanism still largely unknown.

Neuropeptides are a diverse set of endocrine signaling molecules in animals (Zhang et al., 2015; Egekwu et al., 2016; Wang et al., 2018; Li et al., 2020). Recently, many studies have focused on the identification of neuropeptides and their roles in physiology and behaviors of decapod crustaceans, such as in *Scylla paramamosain* (*S.paramamosain*) (Bao et al., 2018), *Carcinus maenas* (Jodi et al., 2018), *Chorismus antarcticus* (Toullec et al., 2017), and *Litopenaeus vannamei* (*L.vannamei*) (Zhang et al., 2020). Existing studies have demonstrated that neuropeptides play important roles in alleviating environmental stressors, such as ammonia (Si et al., 2019; Zhang et al., 2020), low salinity level (Barman et al., 2012; Li et al., 2014; Liu et al., 2014; Sun et al., 2020), hypoxia (Sun et al., 2020), and low pH value (Liu et al., 2019). However, there has been limited information regarding to the neuropeptides and their regulatory roles in ammonia detoxification in swimming crab. The identification of neuropeptides represents the first and essential step to elucidate the functions of these molecules against ammonia stress.

*In silico* transcriptome mining is a powerful tool for identifying neuropeptide repertoire (Christie et al., 2008; Saowaros et al., 2015; Nguyen et al., 2018). In the past several years, many neuropeptides have been identified in crustaceans, using the second-generation RNA-seq technology (Veenstra 2015; Wang et al., 2018). With the rapid advancement in transcriptome sequencing, third-generation sequencing, including Oxford Nanopore Technologies (ONT) and Pacific Biosciences (PacBio), has been increasingly utilized in transcriptome analysis in crustaceans (Deamer et al., 2016). It has significant advantages in read length, accuracy, transcript identification, and genetic information richness, compared with the second-generation RNA-seq technology (Abdel-Ghany et al., 2016). In the present study, we explored the putative neuropeptides in *P. trituberculatus* using long-read ONT sequencing method, which analyzed the variation of genetic expressions of neuropeptides under the ammonia stress condition. To the best of our knowledge, this is the first report of nanopore transcriptome analysis in the swimming crab. The dataset of this study will lay the fundament for unraveling the regulatory roles of the neuropeptides in ammonia toxification process, and provide a valuable resource for genetic studies in this species.

## Materials and Methods

### Animal and Sample Collection

A total of twenty female swimming crabs (202.6 ± 9.8 g) were obtained from Haifeng Company (Weifang, China), and acclimated to laboratory conditions for 14 days. During acclimation, the crabs were divided equally into two groups and they were cultured in different 3000-L tanks, water temperature was kept at 18.2°C ± 0.5°C, aeration was applied continuously, pH was 7.6 ± 0.2, the salinity was 30.3 ± 0.3, ammonia-N concentration was below 0.10 mg/L. The swimming crabs were fed *ad libitum* with live Manila clam *Ruditapes philippinarum* daily, and the feed residues were removed before the next feeding time. One-third of the rearing water was exchanged daily. After acclimation, three individuals were randomly chosen for both control and ammonia exposure groups. The control group was reared with low ammonia level (ammonia-N < 0.10 mg/L), while the exposure group was reared at high ammonia level (ammonia-N = 20 mg/L). The ammonia-N concentration for treatment group was realized by infusion of calculated amount ammonium chloride (NH_4_Cl) stock solutions which was prepared with filtered seawater, and checked using salicylic acid method with spectrophotometer. Following 24-h exposure, the crabs in different groups were placed in an ice bath for anesthetization for 5min, and then sacrificed for eyestalk dissection and cerebral ganglia collection. The tissues were immediately frozen in liquid nitrogen and stored at −80°C.

### ONT Transcriptome

Total RNA of the samples was extracted with *TRIzol* Reagent (Thermo Fisher Scientific, United States), and RNA integrity was evaluated using the RNA Nano 6000 Assay Kit with the Bioanalyzer 2100 system (Agilent Technologies, United States). An equal amount of total RNA from each sample of both groups were pooled together. Oxford PromethION 2D amplicon libraries were prepared according to the Nanopore community protocol using library preparation kit SQK-LSK109, and sequenced on R9 flowcells to generate fast5 files. All the generated fast5 reads were then basecalled in guppy v3.2.10 with the default options to produce fastq files. The clean reads were filtered using Nanofilt v2.5.0 with options of length = 300. The full-length transcripts were identified using the method of Pinfish pipeline, detected using Pychopper, and aligned to reference genome of *P. trituberculatus* with minimap2 v2.16 (Deamer et al., 2016). At present, there are two chromosome-level reference genome for P. trituberculatus, which are publicly available (Tang et al., 2020;Lv et al., 2021). In order to find more neuropeptide-encoding genes, we used the recent version with higher number of annotated genes (Lv et al., 2021). The consensus sequence was obtained by clustering according to the results of comparison, and known and novel transcripts were identified using Gffcompare v0.11.2.

### Gene Annotation and Classification

The functional annotation of identified transcripts was performed using a Blast search against the NCBI non-redundant nucleotide sequences (Nt), NCBI non-redundant protein sequence (Nr), Gene Ontology (GO), Kyoto Encyclopedia of Genes and Genomes Ortholog database (KO), Orthologous Groups (KOG/COG), Protein family (Pfam), Clusters of a manually annotated and reviewed protein sequence database (Swiss-Prot) with a cutoff E-value of 10^–10^.

### Identification of Neuropeptides

To identify the neuropeptides in *P. trituberculatus*, we searched sequence annotation file for the keywords of known neuropeptides in other decapods. In addition, we carried out a local tblastn analysis with Bioedit 7.0.5.3 software, using the known crustacean neuropeptide precursors which were primarily from *Nephrops norvegicus* (Nguyen et al., 2018), *Lysmata vittata* (Bao et al., 2020), and *S. paramamosain* (Bao et al., 2018), as query sequences. All the identified neuropeptide sequences were validated with Blast in NCBI. The structure of the mature neuropeptides was predicted using a well-established workflow (Veenstra 2011; Nguyen et al., 2016; Bao et al., 2020). All the deduced precursors were analyzed for the presence of a signal peptide using the online program SignalP 5.0 (http://www.cbs.dtu.dk/services/SignalP/). Prohormone cleavage sites were identified based on the information presented in Veenstra (2000). Multiple sequence alignment of the predicted peptide sequences was conducted with ClustalX, and then the sequence alignment file was exported to LaTEX TexShade for conservation calculation.

### Expression Profile of the Neuropeptides Under Acute Ammonia Stress

Total RNA of the samples from the control and ammonia exposure groups were reverse-transcribed to cDNA using Evo M-MLV RT Kit with gDNA Clean for qPCR II (Accurate Biology, China) following the manufacturer’s instruction. The PCR reaction was run on the ABI 7500 fast Real-Time PCR System (Applied Biosystems, United States) with SYBR Green Premix Pro Taq HS qPCR Kit II (Accurate Biology, China). The PCR reaction was carried out in a total volume of 20 μL, comprising of 10 μL of 2× SYBR Green Pro Taq HS Premix II, 0.8 μL each of 10 mM each primer, 2 μL of diluted cDNA, and 7.2 μL DNase-free water. The PCR program was set as followed: 95°C for 30 s; 40 cycles of 95°C for 5 s and 60°C for 30 s. *β-actin* was amplified as an internal control housekeeping gene which is commonly used in ammonia stress studies (Feng et al., 2010; Zhang et al., 2021), and the relative expression levels of the neuropeptides were calculated using the 2^−ΔΔct^ method (Livak and Schmittgen, 2002). Difference in expression level of a specific neuropeptide between the control and ammonia exposure groups was analyzed using independent-samples *t*-test with SPSS 24.0. Differences were considered significant if *p* < 0.05.

## Results and Discussion

### ONT Sequencing and Annotation

**FIGURE 1:**
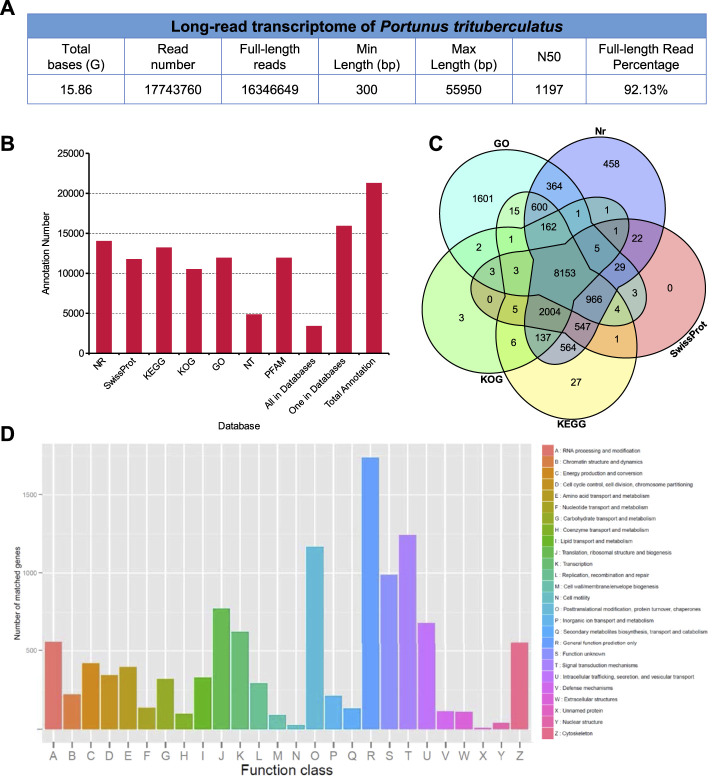
*In silico* transcriptome analysis in *Portunus trituberculatus*. **(A)** Long-read transcriptome of *P. trituberculatus*. **(B)** The functional annotation of non-redundant sequences in seven databases. **(C)** Comparison of transcripts present in different datasets, including GO, Nr, KOG, SwissProt, KO and KOG. **(D)** Functional classification of assembled transcripts.

In order to identify the neuropeptides in the swimming crab, a library was prepared from pooled RNA extracts of eyestalk and cerebral ganglia, and sequenced using the Oxford Nanopore PromethION platform. After removal of low quality reads and adaptors, a total of 15.86 GB clean reads were obtained (Figure 1A). After filtering rRNA, reads with the primers at both ends were considered as full-length reads. A total of 16,346,649 full-length reads were obtained, accounting for 92.13% of the clean reads (Figure 1A). Comparison of clean reads with the reference genome resulted in a mapped rate of 73.81%. The mapped reads were clustered to obtain consensus sequences, and the consensus sequences were aligned to reference genome of *P. trituberculatus* to eliminate redundant reads (Lv et al., 2021). After removing redundant reads, 25,054 non-redundant sequences were generated. The non-redundant sequences were functionally annotated in seven databases (Figures 1B–D). 14,014 (65.92%), 4,843 (22.78%), 11,912 (56.03%), 11,746 (55.26%), 11,912 (56.03%), 13,195 (62.06%), and 10,487 (49.32%) transcripts were annotated in Nr, Nt, Pfam, Swiss-prot, GO, KO, and KOG database, respectively. 3374 (15.87%) transcripts were annotated on all the seven databases. Generally, ONT sequencing generated a set of high-quality transcripts for identification of neuropeptides as well as other genetic studies in *P. trituberculatus*. All the reads of ONT sequencing were deposited in the Genome Sequence Archive (GSA) of the China National Center for Bioinformation (CNCB) with the accession number CRA006434.

### *In silico* Mining of Putative Neuropeptides

Based on Nr-annotation and homology searches, a total of 51 putative neuropeptide precursor transcripts were identified in ONT transcriptome of *P. trituberculatus*, including isoforms of Adipokinetic hormone/Corazonin-related peptide (ACP), Agatoxin-like peptide (AGLP), Allatostatin (AST), Bursicon β, Calcitonin related peptide (CRP), CCHamide (CCH), Crustacean female sex hormone (CFSH), Corazonin (CRZ), Crustacean cardioactive peptide (CCAP), Crustacean hyperglycemic hormone (CHH), Diuretic hormone (DH), EFLamide (EFL), Eclosion hormone (EH), Ecdysis triggering hormone (ETH), Elevenin, FLRFamide (FLRF), Glycoprotein hormone beta-5 (GPB-5), GSEFLamide (GSEFL), HYGSLYRamide (HIGSLYR), Insulin-like peptide (ISLP), Ion transport peptide-like (ITPL), Molt inhibiting hormone (MIH), Myosuppressin (MYO), Neuroparsin (NP), Neuropeptide F1 (NPF1), Pigment dispersing hormone (PDH), Phoenixin (PNX), Prohormone, proctolin, pyrokinin, Red pigment concentrating hormone (RPCH), SIFamide (SIF), short Neuropeptide (sNPF), Sulfakinin, Tachykinin, Terminal ampullae peptide (TAP), Trissin, and Vasotocin-neurophysin (VNP). These neuropeptides cover most of the previously identified neuropeptides in other decapods (Bao et al., 2015; Nguyen et al., 2018; Bao et al., 2020), and the majority of the neuropeptide transcripts (86.3%) contain the complete coding sequences, which indicate that ONT transcriptome sequencing is a powerful approach to mine the putative neuropeptides in the swimming crab.

**FIGURE 2:**
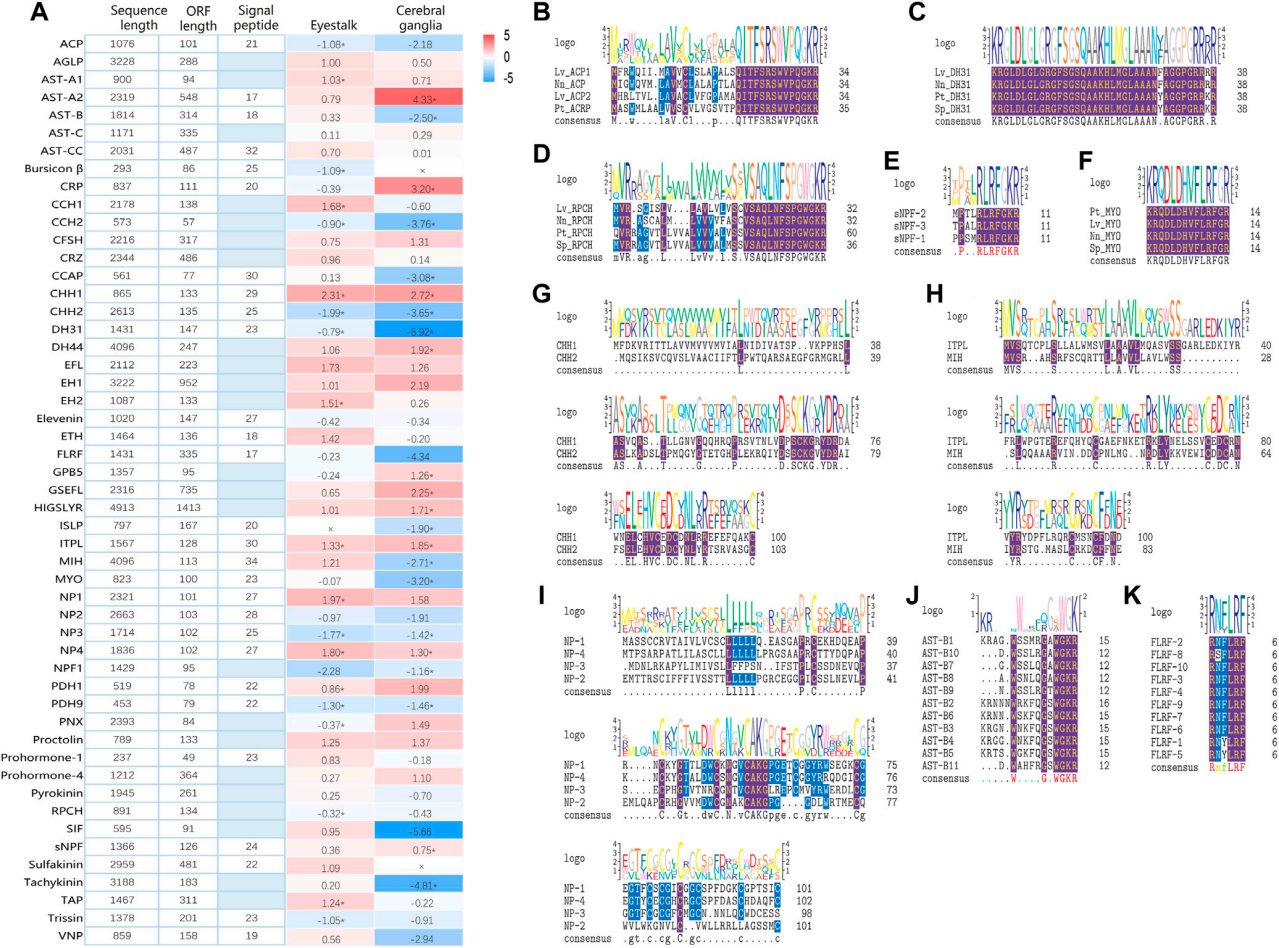
Overview of putative neuropeptides identified in *Portunus trituberculatus*. **(A)**. Sequence length, ORF length, and signal peptide in each putative neuropeptide. Shades of blue indicate an absence of the signal peptide. Gene expression profile in eyestalk and cerebral ganglia are transformed into log2-fold changes (right) with color-coded as described in legend, and the asterisk indicates differentially expressed genes (DEGs) under ammonia stress. Sequence alignment of ACP **(B)**, DH31 **(C)**, RPCH **(D)**, sNPF **(E)**, MYO **(F)**, CHH-I **(G)**, CHH-II **(H)**, NP **(I)**, FLRF **(K)**, AST-B **(K)**, and AST-B **(J)**. Conserved amino acids are shown in purple shading while similar amino acids are shown with blue shading. Abbreviation: ACP, Adipokinetic hormone/Corazonin-related peptide; AGLP, Agatoxin-like peptide; AST-A, Allatostatin A-type; AST-B, Allatostatin B-type; AST-C/CC, Allatostatin C/CC-type; CRP, Calcitonin related peptide; CCH, CCHamide; CFSH, Crustacean female sex hormone; CRZ, Corazonin; CCAP, Crustacean cardioactive peptide; CHH, Crustacean hyperglycemic hormone; DH, Diuretic hormone; EFL, EFLamide; EH, Eclosion hormone; ETH, Ecdysis triggering hormone; FLRF, FLRFamide; GPB-5, Glycoprotein hormone beta-5; GSEFL, GSEFLamide; HIGSLYR, HYGSLYRamide; ISLP, Insulin-like peptide; ITPL, Ion transport peptide-like; MIH, Molt inhibiting hormone; MYO, Myosuppressin; NP, Neuroparsin; NPF1, Neuropeptide F1; PDH, Pigment dispersing hormone; PNX, Phoenixin; RPCH, Red pigment concentrating hormone; SIF, SIFamide; sNPF, short Neuropeptide; TAP, Terminal ampullae peptide; VNP, Vasotocin-neurophysin. Lv, *Lysmata vittata*; Nn, *Nephrops norvegicus*; Sp, *Scylla paramamosain*; Pt, *Portunus trituberculatus*.

As shown in Figures 2B–K, highly conserved motifs were identified in a number of neuropeptide families, such as AST-B (XWXGXWamide), FLRFamide (R/K-N/S-F/Y-LRFamide), MYO (QDLDHVFLRFamide), RPCH (pQLNFSPGWamide), sNPF (XPXXRLRFamide), and family of CHH and NP (conserved Cys) (Backing et al., 2002; Christie et al., 2010; Christie 2014; Nguyen et al., 2016; Liu et al., 2020; Qiao et al., 2020). In addition, the amino acid sequences of all the putative neuropeptide precursor and their structure information, including sites of bioactive mature peptides, location of cleavage sites, and precursor size were provided in Supplementary File S1, S2.

### Expression Profile of the Neuropeptides Under Ammonia Stress

Although it is well-established that neuropeptides are critical in regulating stress responses in crustaceans, limited information is available on their roles in defending against ammonia stress (Si et al., 2019; Zhang et al., 2021). In the present study, we investigated the expression profile of neuropeptides in the swimming crab under ammonia exposure for the first time. As shown in Figure 2A, 19 and 22 neuropeptides exhibited differential expression in eyestalk and cerebral ganglia, respectively, after ammonia stress. In eyestalk, 9 neuropeptides showed upregulation under ammonia stress, while 10 exhibited downregulation. In cerebral ganglia, 10 and 12 neuropeptides were upregulated and downregulated, respectively. Among these differentially expressed neuropeptides, 6 including *CCH2*, *CHH2*, *DH31*, *ITPL*, *NP3*, and *PDH9*, showed the same expression pattern in eyestalk and cerebral ganglia.

It is well established that CHH regulates a diverse array of physiological processes in decapod crustaceans (Chen et al., 2020). A recent study demonstrated that CHH is crucial for regulating ammonia excretion in the white shrimp *L. vannamei*. *CHH* knockdown can significantly downregulate ammonia transporters in branchial epithelium, and result in ammonia accumulation in the hemolymph of white shrimp (Zhang et al., 2020). In addition to modulating ammonia excretion, CHH plays a major role in stimulation of glycolysis and lipolysis, which can result in higher levels of hemolymph glucose and ATP in decapods (Backing et al., 2002). In this study, a significant upregulation of *CHH1* in eyestalk was observed after ammonia stress. Given that ammonia excretion is highly energy-consuming, the result indicates that CHH1 may coordinate the processes of energy metabolism and ammonia excretion in the swimming crab, facilitating the defenses against ammonia stress. Interestingly, the other type of *CHH*, *CHH2*, was significantly downregulated. This result indicated that different types of CHH have distinct roles in regulating stress response to ammonia. Further studies are required to reveal the detailed functions of the different types of CHH under ammonia stress.

Our recent study found that ammonia exposure can result in a remarkable reduction in vitellogenesis in the swimming crab (Meng et al., 2021). In accordance with that result, expression of the key neuropeptides in vitellogenesis regulation, including *NP1* and *RPCH*, significantly changes after ammonia stress. *NP1*, functioning as inhibitory factor of vitellogenesis, has showed significant upregulation, whereas *RPCH* which promotes vitellogenesis was downregulated after ammonia exposure (Bao et al., 2015; Nguyen et al., 2016; Nguyen et al., 2018; Liu et al., 2020). Taken these results together, the neuropeptides *NP1* and *RPCH* may mediate the ammonia-induced inhibition of vitellogenesis in the swimming crab, which could represent a tradeoff between the allocation of energy to ammonia-defense and reproduction.

In summary, we identified putative neuropeptide-encoding transcripts from eyestalk and cerebral ganglia of *P. trituberculatus* using long-read ONT transcriptome sequences for the first time, which they were analyzed for their expression profiles under ammonia stress. This study provides a fundamental support for future research on the roles of neuropeptides in ammonia stress regulation process, and a valuable dataset for genetic studies in the swimming crab.

## Data Availability

The datasets presented in this study can be found in online repositories. All the reads of ONT sequencing were deposited in the Genome Sequence Archive (GSA) of the China National Center for Bioinformation (CNCB) with the accession number CRA006434, PRJCA008798.
