# Supplementary material for: Identification of Neuropeptides Using Long-Read RNA-Seq in the Swimming Crab Portunus trituberculatus, and Their Expression Profile Under Acute Ammonia Stress
Source: Front Physiol. 2022 May 16;13:910585. doi: 10.3389/fphys.2022.910585 (PMC9149262; doi:10.3389/fphys.2022.910585)
Supplement: Supplementary file 2 [file Table1.DOCX]

**Supplementary file 1. The amino acid sequences of all the putative neuropeptide precursor and their structure information**

Purple and yellow highlight represents signal peptide and putative cleavage site(s), respectively. The letters in orange and purple are mature peptides and conserved Cys site, respectively.

**Adipokinetic hormone/corazonin-relatedpeptide(ACP)**

MASWMLAALVVSCVLVGSVTPQITFSRSWVPQGKRSSPTGDIPEPLDPCRDARAATLSSLAGHLLDMMNDVAAADHRPLPDDGTTALRLRNAMMDRRRRVA

**Agatoxin-like peptide (AGLP)**

MYRCRGLVRRVPNSCRTADCVTKHGKKTVNPSRGYADLPYKPPSMNELPKPQGSWSQRHERIQRKYNLQLAAGIGFSTFTFFVLKSSGLVDFGLNPGQIQLPKSDSAAAAGGEEEEEEESSVAEASAEVIEEEAPAAVEEAPAAVEEAPAAVEETPAVVEEAPAAVEEAPAVVEEAPAAVEEASAPVEEAAPAPVEETVPAPVEEAPAAPVEEPPAAPVEEAAAAPVEAAPVEEAPAAPAEEAPAAPVEEAPAAPVEEAPAAPVEEAPAAVEETPVAEAAPAASTEGS

**Allatostatin-A1(AST-A1)**

MTAGLSSAGFSSSALLCTILGLLLFLLLLLLFLLLHLLLRLVLLLLLLFLLLRLLLGVFFLCFLLRRLLLHLLLHLLLRLLLLGLLGDLDFSGV

**Allatostatin-A2(AST-A2)**

MGVRVLVFLVAAAAVAMAGYIPPTKSFGAGASAGSKASASSSSSSFGSASAGASSGAGSFGSAGSHGAAGGHGSGGSFGSAGGSAGAHGTGSSFGSAGAHGAGSSFGSAGGSAGGSAGTHGAGGSFGSTGGSAGAHGAGGSFGSAGGSGGAHGVGGSFGSAGGSAGGSAGAHGAGGSFGSAGAHGAGGSAGAHGAGGSFGSTGGSAGGHGAGSSFGSAGGSAGAHGSGSSFGSAGGHGAGSSFGGGAGGGAGGHGTPHGTGAGCCGAASSGSGTSHGAGGSGSSTSHGTSSFGSGTSHGAGSSGFGTSHGTGSAGSGASHGAGASGSSTSHGTSSFGSGTSHGAGSSGFGTSHGAGASGSGTSHGTGSSHSSGAFGASGSSHGSGAFGAGGAGHSSGAFGAGGAGGAGHGSGAFGAGGAGGAGHGSGAFGAGGAGSGSGAFGAGGGAGHGSGAFGAVVQVVQATVLVHLVLVVLVLVLVPLVLVVVQAMVLVPLVLVVVQAMVLVHLVLVVLVLVLVPSVLVVQVLVPGPSVLELTGPQEALELAGRR

**Allatostatin-B type(AST-B)**

MQLATLTATLLTLVAAAAAQDEGSGLAVAQAKRAGWSSMRGAWGKRDDSSDQGLQVSEDKRNNNWRKFQGSWGKRGEEIQDAEDKRGNWNKFQGSWGKRADDITEEAALQAAEEKRGGWNKFQGSWGKRGDEVASEDDLQDAEDKRTSWGKFQGSWGKRQDDLIQLQDLEDKRNNWSKFQGSWGKRAGWSSLQGAWGKRAWSNLQGAWGKRSPNDSEDIDDEALEEEELQVSPEALARMVAASPVKRGWALWGKRPDYPAVSPRSTNWSSLRGTWGKRSGDWSSLRGAWGKRVPNDWAHFRGSWGKRSPDTLIA

**Allatostatin-C(AST-C)**

MAKVKVVKVEETGKDVGTKASDKKNKNEKKILKVISKRNGTDNASVPKKKKRNRGKKNKSNTNGIEASPETKNGESSPTIDEENSEPTNDISSSSVEKPMKEEPATTTSETPEAATKTTESGESEGPNTEGKKKRKRKNKRKASEALEGASESTDNKEDGSEDTAEGAKGIKRMKVEEEKDDAEEEKPKKKTKKMKAEKKGEDDAEGQSASKEKKQQQEKKYVLFMGNLPFELTEEEVREHFKIVADNIVRVTLLNRKKSGKSKGYGFIELKDADSYNKSLKLNNSQLKNRKIHVQFTTPGKKTKARKALIKNKTKKLLGQKKGKKIKKEGGRKH

**Allatostatin-CC(AST-CC)**

MATTTTTTTTTRTLLLLLLPLLLLLLQATTCHASDFNDIYDYEYSYGDLGYSYDDGEAKKREKEGDDALQIASHVADTGSSIGGLSDLPQIPLCCPLNAVYEMGKNCTTAPTDWTWRPHLSGEPNANFMYTGFPKCKVNEPVPFYQKEVFFNDGEAFVPNYVQLHSIPKEKYCVSKVYDKTDPEQCEIHRTKVFVCLDKEQEEPGLYWTGVSLAHTFLALTLCTFFLVRDLRCLQGQYMICFLISLLAYNICLLPGSVIVLSISFVSCVSLGAVKYFFFCGVMLWFNVICFDVWRTMQSKQDIGSRRRFLLYSVYTWVVGAVLTTAVLVVPYLSGKTHDSTLMETNTDDCKLKTDINMILQLVEVLLMVVNLVFLILASTHICKYPKFGEGLPRCDATLSLKQGWKLYIIMIGHTLIDVPDDILTIKVVDLWRYVLIESMALFAVFAYRKTVLRHLYSVFYCGRHVPRSDDDANGGIPEKHQLTNMH

**Bursicon β**

MWCGRGLLAAAVVVVVAVLLPNTVHCRTYGIECETLPSTIHISKEEYDDTGRLVRVCEEDVAVNKCEGACVSKVQPSVNTPSGFLK

**Calcitonin related peptide(CRP)**

MHHFYIGLCLVVLVCVSGNAHPVDYESDEAFLSEKMREYLMLRKLLINALSERTPLKESPRKRSCYLNGLLSHGCDYQDLVSSTVEKNYWDSLNSPGKRRRRRREAQEEEI

**CCHamide-1(CCH1)**

MSPKMEGLSGESSEESSHSGMSSRRIVKNTEEYRKRRERNNQAVKKSRLKTKKKTQEMVDRVTQLRNENEELEENIKILNKELGLLKGLFLEHAGNAHGVRLNEAELARMLDEDLEVDKGVALLMNLSQGSSPQNKWN

**CCHamide-2(CCH2)**

MLPVRPLVFRGARKARRRAVWQPGPRRSVPALQPAGGGGRGALRTGGGAGHQSSQHG

**Crustacean female sex hormone(CFSH)**

MWSSPEYLKRKDEKPQISAKVPWSRSKGTNKEQLPNTSSRGPVMMEPLYQSVRSNPKKYQGRVPSIVALLSHHAKSHQKTQKSALQSELPTTKIFEDSSENTMWQQNRFAPVSQEIHRAMAREEETIFDTSKKQKVDFNKGGGYSFSYHLSPSTMPASPSRPSVLNLPPPPQMPLPPVPEVHDYAEPKPGPSSRGETGLTPPKPSVSWKEFQSSTATKVHKMYQPVAYKAQRLSESVSFLNPYTRRTECETSAEDKGSSQIIVTPHSEKQRRESTSLFLPDDDLVKPKDIDQQKLSTVLVLSLFVIALIIFYLVYFL

**Corazonin(CRZ)**

MPQDGVGSKSGGKKRSGGVPRVRSHTDAELQYYRDQDHDVLPSHLRVRLHDLFLQIEKEFEVLYTDNLALQEKVDSLTERLERESVAGERVTGTEETDGASTKGGIKTKSGGPSSQKVKAANKLRVQTSKIVSSFKNDSVSCRMVKEYVGHRDGVWDVAVSRGGTPVIATASADQTACVWGIDGGKNLLQYTGHSGSVNSVKFHPSKDLILTSSGDHTAHIWQAVITPDQLRVHSSEDEVDGSDREDEDDGGRCDGSGGPATLRTPLRELMGHTSVVIAADWLPGGDQAITAAWDRTANLYDAHTGELLSQLIGHDQELTHCCSHPTQRLVVTASKDTTFRLWDFRETIHSVSVFQGHTESVTSVCFTREDKVVSGSDDRSVKVWDVKNMRSPLATIRLDSPVNRLAVSPTNVIAIPHDNRHVRLYDLAGSRLARLPRSNRQCHRRMVCSVAWGDDSLGARCNLFTAGFDRVVYGWSVHSDKEGKD

**Crustacean cardioactive peptide(CCAP)**

MYFTSLSGRAGLIIAGTILLMAFLIADTEGGTVAKRDIDSLLDGKMKRPFCNAFTGCGKKRSDPELEGLAFCHSSMT

**Crustacean hyperglycemic hormone 1(CHH1)**

MFDKVRITTLAVVMVVVMVIALNIDIVATSPVKPPHSLASVQASTLLGNVGQQHRQPRSVTNLVDPSCKGRYDRDAWNELCHVCEDCDNLRREFEFQAKCRDGCFASEVFVDCLYDLGKDVDLYLEMASSLRG

**Crustacean hyperglycemic hormone 2(CHH2)**

MQSIKSVCQVSLVAACIIFTLPWTQARSAEGFGRMGRLLASLKADSLTPMQGYGTETGHPLEKRQIYDSSCKGVYDRAIFSELEHVCDDCYNLYRTSRVASGCRENCFENDLFEECVFELMLPDEMFLIRDAIRG

**Diuretic hormone 31(DH31)**

MNNFSVVFASLVAAFVLLSSVHATPINREPSRAVVEIDDPDYVLELLTRFSNSIIRAKELEKFVRSSSGTKRGLDLGLGRGFSGSQAAKHLMGLAAANYAGGPGRRKRESQAAPLSLHHDDHPAAQEHAAIAAAAAATAGLQQHSSR

**Diuretic hormone 44(DH44)**

MSHLGLADLGLAAFTGHQAASPTENGFWPSTSPSQAASPLATSPPDPWGGLGQSPPEAWEMFSRSPNTAFLHSCQSPPSSSMFPTTLFSPPHPIQSPSPIQSPPPPVHSAPQVVQSDLKSHPPTVQSPSDTNPFPFPTFSTTGEPNDPFSIKLPQQKCHNNEQLPSLPLSPVSPVSPNGNENPFLSTTNPFIAPPTPLKEMANSSPQPPVECPRVLVASGSSGCSPGRPVWTADLFSGSKNKDVICS

**EFLamide**

MNYSSCNSTTSVAAPPRRGALIVIEGCDRTGKTTQAMKLVEYLNGSDRKAIFMRFPDRTTPIGCLIDSYLSRGVNLEDQVIHLLFSANRWESHPKIISTLKSGASVIIDRYAFSGVAFSAAKESLGLQWCKGSDAGLPKPDVVLFLDLPLEQAATRGEYGSERYEELKFQQRVYKNYMALRDDTWKMIDASKSIEEVQRSMEAEVERVMKESKFGPLPGLWWS

**Eclosion hormone 1(EH1)**

MEEEQELAKMEITKENGLAQEEDDKPQPPPPTPPPEQNNTHTHERPHLPTIDFVLVYKPSPTSNTTTTTTTDTTTVATLDKDEEKHEMWRKNFESELRTEGLILEEDSVDELPLRFVKIHAPFEVCRRYAEILKLRMPMREKFDMRQGNHRVILHFPGLPALEFTPPAVFQDMTDCVSRVFNFVRLDPSVFPPRNRNFTAVYARDKDYLFEVTPEFFRPSVRARIIDFILRRKRYGGREEEDKEEEEDVKTGGRGRGGEEGRAIHGTETETFEFGIEKLLAEGAYLAAYPLHDGDLHSNTSTTTNQRALLRENWASLRKFYKYQPLDHIKDYFGVKIGLYFAWLGFYTYMLIPASIVGIFCFIYAVSTISEHAPSNDICHEHNNMTTMCPLCDKFCDFWDLRETCFHAKFTYLVDNPSTIFFTAFMSLWAALFLEMWKRYSAEISHRWDLTGFDIQEEHPRPQYLARLAHIKKKTVNIVTKTVEPRPPFWRMRFPGVILSLSTVFLLVTMALAAVLGVILYRMSVLAALSIHGESVLTSYALIFTSTTAACINLVCIMIFNQLYARLAERLTELELQRTQTEFDDSLTLKIYLLQFINYYSSIFYIAFFKGKLIGRPGDYKRVMGFRQEECSPGGCLLELSVQLAIIMVGKQAMNTVLEMVLPLLYKWYNFLTVGEGLREAYVNWPRWAKDFRLVNFDPRGLFPEYLEMVLQYGFVTIFVASFPLAPFFAFLNNIFEMRLDANKLLSHFRRPIPQRVKDIGVWFKILDSIGKLAVITNAFIIAFTSNFIPELVYRYVVSDRNSLDGFLDYSLATFKVTDYPAAYRPDSPTPPDYCRYPDYRQPPDSPTPYDYAPIYWHILAARLAFVVVFENVVCVIIMFIKWVIPDMPFRLQEQIRREAYVTNEIIVEQELRRAREGGEDLSGTPLRPNRTRRRYRGASWGGEPSKGEVQV

**Eclosion hormone 2(EH2)**

MCRGSRWLTASRRTLSPRPAPASRPPRTPAPHARCSGIPSCSAHTTTGRNQATAGREESSPGRLKSTTFLKHRAQTALETGLLQFEQSPTSVYLHQNQVSREEMIRLQRCARRRDSWNRPSHNRDWGGGGGTL

**Elevenin**

MASTAPRTCLVLHTILLLTALASFASCGQAIDCRRFVFAPQCRGIIAKRTVSDSAIGLPDALQEQRQWSESIPLDYVVPEYSPAYQRVRSTYTRPSAASRDAAVGAQFSSPNAAMTEMGVRDSGDLELLSPYVKIIHRSEKLPYERK

**Ecdysis triggering hormone (ETH)**

MAWLVAATVLAAVVSIASADAGHFFAETPKHLPRIGRRGDLPPLTTLLSEEDARSSGAGTRSMTEALAGLDSDGDGCIGVAELLRIPAVRVALLLQNPALLTPANLATPEVDAHATEDTFASDRRPEPRLLRYLQK

**FLRFamide**

MIAVAWVLLSGVAWCLASPLTPVPGAIEASPSTHNAPGSDIPEVPQEKRLLKYFLPSSSSWVPTQQEGSKRGYSRNYLRFGRSEEDKRGGRNFLRFGRADISSIEDTDMLPETEDSMEKRNRNFLRFGRDRNFLRFGRSDAEEFGLPGGPLAFSNLQEDDTEDYPVEEKRAGHRNYLRFGRGNRNFLRFGRDDNRNFLRFGRSVDRQLKEQKVHEAPLAPTTVPHSPAKTQDSHRSKRSASPYSYVVMPSHGPAAWAQDFQPEQEDEDLEVAVDGPEAAVTKRGYNRSFLRFGRDRNFLRFGKRNDDSASDVVVVEPASYPRYQRAPQRNFLRFG

**Glycoprotein hormone beta-5(GPB-5)**

MSFLLQFSLHCLLSEHTTAWPILLFSRDISLSPSCISLLGFLLSFFQEMLCRVFLFPCLSNESKQSEENEEMNEGMFGASEGSLFGGHRGCLDPS

**GSEFLamide partial**

MLPSSGYFRGIACPGQLKGACNRPYCHFRHDMKVSPEKNASPSPSSSQAAVSTPVQEEADAVDEEKLEKIEHQIMSKAVNECSSDSLTPAVTSPSKPEEQQSQPHTSQQQQEPAAASDEVPKSEILQQLMSEALKKVLAENPTLASTVDASSIRIGIDPVTQELSEVNKTKKQLYHRPPDTPSYNPTPIKELKKRKQSVDGVHNLVYNPTCSRTTGDKGSGDEGDVMGEFSSDESEEIEEKNDDFDLLEEVLAEHSKNNKVKVKKKKVKELGKLQYSLSNAHKATVDNNIMITDAKKLSDKEVKKYELPQTESVNKIITDGPYFLPPSPIFTSSSDGFEAFSAKATLQEEETSVPEECVDATSPHTESVKTNSQPATEKKTTSKQTVEQPKSSSQPENAEKVTKASGHAHLPQLLLARGAPKLSRPNIRTWTPLVRNNLLSSETQKTGDQLDEILQLMDGSDPSSKRKLKENEHQTSNKKDSENLRPKKKYKVDLNKEIESLSNPALEHSKESVTPEEPAIQQPEPSDNQDSDRKSTKESIVSDEKEAAKRDSPKESEEKEETDGSKAASMESEDSEGDTLSEESGDDSDYRRSRHKKKRKRKRSRSSSSGSDYSHHRRKKRKRSHKRHHKRSKRSRRRRNSTSSLSDQSQESEEEREEKKNDEDEEEENDDTEEEEENGEEEKENKSKEDSKSEDEEDSNDDIEHTRKSKRHRKKHKYYKKLKHRKSKHKARRR

**HIGSLYRamide**

MIDVTIKTLDSQNRRYSVPDDITVKQFKERIASSVDIPADKQRLIYCGRVLQDDKKLAEYNVHEKVVHLVMRAPPQANSRNGSGGASSSATGSPGSGYHHHHHHHHHHHHHRRQQQPEALPTQGPSSSGVRLHQARTMLDRASAVLDRLDRRLQVQPLASSSPENDTAAGTSQGAEAAAAEAGPMETESNEESQPSSSTGAEEPTPSTSTITPPTEEMEALYSEMDALNNMDPATLIPAPPPPPMMGAINGSLAGGLAQAASAAVASALSSLARGGSSTSSVPTTTTTTTTTQPSTAVSGTLPATASATSAPRAVTTTTTSSGSSRSTGAVPQGSVRSTLTIRITNPWSSNSESSSSTTATTSTTTTSPSNTSNTSRSSSTVRAPAPAEATTSESSRSRRSSGSQDRASPSSSNATAPGPSEGTGGSSSEGGAGMMRLEHPRCAVMVEVLDQYTQIQRRLEPYLARYYQAMANDPVFSDGEADSLQQQQWLLWRVSEVLHFVSHAMHAISDIMVDLRRAPPRQLRARPIIIQQPALVQAQINVTTSSDPSRGGMSVFSSSRPSTTTTSQSSSANQSSSTTASSQTTTTTTTNVSSSTTSSSSSSSSSSSTSTSSMSTNTSADPSSNYLLVSGPVTGVLSATTTTPASSTPSTTSTTTPSATPHPARNLASLLNLGSMQGFPMDGGDLVLMEVGPHGITIDSVSAEGGSTGGSAPPPELIQNLVSSITSQLGMHIGGATPSTTAPASTTSAAASSASVSSSATTANTGVPSAGPGRNSQAAGNSGTNTTNVTQTRVTHRPHVHVTPLNVPGMGMNQFDPFLPCQSHHIRPATRRRHQAQTQTQDGSSPAQSRRRAAGAQAQEGGGSQSAQTRPQVGVSPTASSANPLAVFANLMAEAFGGRQVAAVPQGDQQPQQQQQQQQSSAPDASESQISDQMFAQLVQGVMSQVSGSLNAEGGEGDNQQSGATLHEFLQPFDGGMFSEGEENSLFYQLFNTVALSLSIGDLVQLFFGRSRTVNQLRAPLQRFMREQALQGNQPTEENLNRAIDNILQDLHPHLVLTAGEARVHEGIDYVYTVHNFVRHRLHDIFNLVLNHTDEETFGPSLVSLMRRTVGEFIALSLYCFSDGAQGLERVLQERVRSIMSGVSPAIQNWSINTSIMQLRSMMSRLTITDDYIRRYVVSPDEGRRMEDEHSRRQRANEAANAKPPQRPLAPTTEGSVTGNNVVHVVPANGPSVEPMEVEVGCMEEVTTTGSELELAVEGATGGASEAKEEETLVEEPPIIITDKPQPWHSAVPQDWIPVITRDVERQRRGVPDTGLSDAYLCGMPLKRRKLASQHKPHGSVQQVIQDTLRHCMRGAGINRVVMDSVVGEAASQLGDSFVGHMRTSLRDRLNHNKDFLPEKFPKSEENIRKGK

**Insulin-like peptide(ISLP)**

MKVVVLLLVVVTAMQTGRVRGSPRTLPEGGLVKQGERRLCGWRLANELNRVCKGVYNVPTVSTNALFYLKGRGGKRVDLWPVGGREQQFPSRTHAPADDLRASHLSEPHLFQRPADRPTGERQRLPLLTGAEASQVVVGRSPRVKRGLSAECCRKACSVSELAGYCY

**Ion transport peptide-like(ITPL)**

MVSQTCPLSLLALWMSVLAAAVLMQASVSSGARLEDKIYRFRLWPGTEREFQHYQCGAEFNKETRKLYNELSSVCEDCRNVYRYDPFLRQRCMSNCFDNDMFFKCTDQLMYPIEKIKDYEDMKMQIKA

**Moult inhibiting hormone(MIH)**

MVSRAHSRFSCQRTTLLAVVLLAVLWSSSLQQAAARVINDDCPNLMGNRDLYKKVEWICDDCANIYRSTGMASLCRKDCFFNEDFLWCVRATERSEDMMQLKQWVRILGAGRI

**Myosuppressin(MYO)**

MVFRLQPWCSLLLVGVVVVLGVCAGVGETIPPPICFNQKLVLTPYARRLCAALNDISKFSRAMEDYLDAQAIKNSMGVNEPEVKRQDLDHVFLRFGRAQQ

**Neuroparsin 1(NP1)**

MASSCCRVTAIVLVCSCLLLLLQEASGAPRCEKHDQEAPRNCKYGTTLDWCKNGVCAKGPGETCGGYRWSEGKCGEGTFCSCGICGGCSPFDGKCGPTSIC

**Neuroparsin 2(NP2)**

MEMTTRSCIFFFIVSSTTLLLLLPGRCEGGPICSSLNEVLPEMLQAPCRHGVVMDWCGNAKCAKGPGGDLWRTMECQWVLWKGNVLCVWLLRRLLAGSSMCPG

**Neuroparsin 3(NP3)**

MDNLRKAPYLIMIVSLLFFPSNIFSTPLCSSDNEVQPSECPHGTVTNRCGNTVCAKGLREPCMVYRWERDLCGGGTFCGCGFCMGCNNNLQCWDCESSAAGR

**Neuroparsin4(NP4)**

MTPSARPATLILASCLLLLLLLLPRGSAAPRCTTYDQPAPKNCKYGTALDWCSNGVCAKGPGETCGGYRRQDGICGEGTYCECGHCRGCSPFDASCHDAQFC

**Neuropeptide F1(NPF1)**

MRGVDGRGGGGGVGGGGLGGAAPQQTGGRGAGCIPGAARGGAGWHTELRGGALSQQAQRLQVQGRTAPLPRRPQCLLRHRWEAEIREARRTSTTT

**Pigment dispersing hormone1(PDH1)**

MRSAVVVAVLVVVALAALLTQGQELKYQEREMVAELAQQIYRVAQAPWAAAVGPHKRNSELINSILGLPKVMNDAGRR

**Pigment dispersing hormone 9(PDH9)**

MRSGVFVAVLVLLVLAALLTQGQQLHVPEREAVASLAARILKVVHAPQDAAAGLPHKRNSELINSLLGISALMNEAGRR

**Phoenixin(PNX)**

MHKTQPAAQSMKIVLSLPLLCPHSINSLASIRDEINNMLSITFCIILYSCYVLFPLIYNVCLEATHCSKYSEHEGSRYHKLYLQ

**Proctolin**

MTLPLNKPFEVSGHQEKDYGAEMGMMRRAPTATSSHPLEESERTYHQRQDQHKLNSATRAFGIGYALHFKHERAAAGCHQIGHLGFLPRSNAHLQALTGRDLELDFTDTLGVQPEVTYNPHLYMEQEQRKSMF

**Prohormone-1**

MSPRLSTVLILAVVVLAALGTTSAKPLGEQDPSAGGPPAFTAREAQVYE

**Prohormone-4**

MFHAATLRHQFEGLRSNMTYEGRLMALVGPHHWPYTLLNFTVTSPTLLTGLSLGQRAHPNRPRSSTADLDAITVSVTLRWSSTPFLKGNTDGEIVQPTGYRLRVWDQKKLVTQIIIEASPHSQDCEAAVGALELDSVYKATLDAILPDGEPGVSLTFSLDAGALHVAGGGLTTDGVSECYSSLQVRCPGSERCVSPYWICDSAPDCPDGVDELGCDTTLCDGFQCWEDVCIPWAWRCDGQPDCKGGDDEYACPSCEEGELQCPWGGPCLQRNATCDGVDHCRDGWDESVALCGSVTCKPGELQCLGGSRCVPHDWLCDGVSDCPAAEDEDATFCSAFNTFKNNLVFNSTNESPSPCEE

REAPKK

**Pyrokinin**

MRYQTIPERTSRPLRIMTPGVPKRLYFAPRLGKRSPSQVESLDERGRRDATSYKEDPEDAVTIPYSWWWPSVSVRRSNFSPRPGKRGEGEIDLPYDYYDPEDDEDEETEDEDEDDGVLQDKRDSTFAFSPRLGKRVQNAAFAFAPRPGKKDNFAFAPRPGKKAGTNFAFAPRPGKRTNFAFAPRPGKKSNFAFAPRPGKKTFAFSPRLGKKADFAFAPRPGKRSDSPSETGDRQTGETWWIGEGGSATVTTQPPFLPPRLE

**Red pigment-concentrating prohormone(RPCH)**

MSLLPFSCVIGSPITSPSLPFLLPQVRRAGVTLLVVALVVVALVSSVSAQLNFSPGWGKRAAAASGNNGGVGEAVSGLHPSVVGAPGGVVPPGSSSSSGDSCGPIPVSAVMHIYRLIRSEAVRLVQCQDEEYLG

**SIFamide**

MNATFREQGTRRLFKPISLFPFVFFLYISVVVVVLVGVQVLLSWWWWRCCVASEGHGARKVSGWELDEVVVLLLLLFLLLLLLVFLFSLSP

**short Neuropeptide F(sNPF)**

MGVNGVKCWVALVCCCLLLCQLTTAAPADYDTLNDMYDLLAVHEVERRAPPSMRLRFGKRDMGWQVSQRSMPTLRLRFGKRNVDEADPILDHHDLIRKDARTPALRLRFGKRGASFGEEDMASQEQ

**Sulfakinin**

MESCKWCLTIALCFSALPALQAQPTPVIQNDAEPPAPVTVDADRQPPFVTVNARQPPPVTLNANDDDDESDVIGDDEREMKLPLRDFIDTNPTEVKGEPVFEGDILLTQDQWRAIRERKGIANEAYRWPKGTDGNVLIPYVFTDDRLNQAAILDGMNHWEQNTCIRFQETTNTDQPHLRFIYGGGCYSYIGMIWFWNGQDISIGNGCDNVGIVSHEIGHAAGFYHEQSRPERDDYVVINWINIDDSKESNFNKASTSEVNNQDTIYDYSSIMHYGSTGFTINGETTIATIDRLAQALIGQREGLSFWDIRLANIMYNCIDKWLDDCNINPDPCQNNGYIGADCACVCPTGTIGTYCETQTGSYYEPLQNSCTENVTTEGTLTSPNHPGNYPEGNCVKWIQAPECHVPMLTFTAFRLYGRNSYCSGNLCCYFDALEIRTDNLDYGEVYCESDIADGTSFTSPTQEMVLFFRTRTELLLGMVC

**Tachykinin**

MYKCVCVCVCVCVCVCVCVCVCVCVCVQTQELSVYINILFIKCIIFRGDHAVVVVVVVVVIVVRGGGRVVKVVAVVVVVVVVVVVVVVVVEVRGGGRVVVVVVVVVVVVVVVVVVVRGGGRIVVVAVVVVVIVVVVVVVAVRGGGRVVVVVVVVVVVVVVVVVVIVVVVVVVVVYKMKVTKRF

**Terminal ampullae peptide (TAP)**

MPVSHTGASSSLVVKFADTEKERQLRRMQQMAGNMGLLNPFVFNQFGAYGAYAQVNVSEQQFMQQQAALMAAASQGTYINPMAALATQMPHAAAPLANGITSPVVPPTSGASQAQPVNGALPSMPSPTMQNFNIAPQPGPNSQPGGSEGAVFANGLPQTFAAPQPIPNGEAATLQPSAAYPGMPYSGVAVYPAPVYGQYAQAPSPQLAPIPHNKVEGCSISGPEGCNLFIYHLPQEFGDAELMQMFLPFGNVISSKVFIDRATNQSKCFGFVSFDNPASAQAAIQAMNGFQIGMKRLKVQLKRPKDANRPY

**Trissin**

MMSMQIYLAMVLAAWAVVVGTCSSSTVSCDSCGPECQTACGTKNFRACCFNFLRRRRSYSLPVRRSQGVGTAAEWKALRGALMSPAAASRGLPFAQFLEASETDPAPQPTKRHRDPSSLASVLTTLLQDSSMDEEDEDEMLELEGEGGGEGIPEASADDATLSRLVAFAFHQPPPPPRSPLNQHYPAHSPPPPPPAGDVGK

**Vasotocin-neurophysin(VNP)**

MQSGVTVTVVVTLLVGSAAACFITNCPPGGKRSGGLMSTLGRARTCASCGPGLLGRCIGPDICCGARIGCFLGSRETRLCRTENMVPITCYNSDLKPCGRMQEGRCAAPGICCTENKCETNDDCVAEDTPAEEVAETQRSGRPRLDLLTAARDRWEEQ
